# A Field Study of Work Type Influence on Air Traffic Controllers’ Fatigue Based on Data-Driven PERCLOS Detection

**DOI:** 10.3390/ijerph182211937

**Published:** 2021-11-13

**Authors:** Jianping Zhang, Zhenling Chen, Weidong Liu, Pengxin Ding, Qinggang Wu

**Affiliations:** The Second Research Institute of Civil Aviation Administration of China, Chengdu 610041, China; Zhangjp@caacsri.com (J.Z.); liuweidong@caacsri.com (W.L.); dingpengxin@caacsri.com (P.D.); wuqinggang@caacsri.com (Q.W.)

**Keywords:** air traffic controller, fatigue, work type, data-driven, traffic safety, PERCLOS

## Abstract

The fatigue of air traffic controllers (ATCOs) on duty seriously threatens air traffic safety and needs to be managed. ATCOs perform several different types of work, with each type of work having different characteristics. Nonetheless, the influence of work type on an ATCO’s fatigue has yet to be demonstrated. Here, we present a field study in which the fatigue of ATCOs working in two types of work was compared based on an optimized data-driven method that was employed to detect the percentage of eyelid closure over the pupil over time (PERCLOS). Sixty-seven ATCOs working within two typical jobs (i.e., from the terminal control unit (TCU) and area control unit (ACU)) were recruited, and their fatigue was detected immediately before and after shift work using PERCLOS. Using a Spearman correlation test analysis, the results showed that the influence of work type on an ATCO’s fatigue had interesting trends. Specifically, the ATCOs at the TCU who handle departures and arrivals, which include converging with and maneuvering around conflicts, retain normal circadian rhythms. Their fatigue was significantly influenced by the various demands from tasks focusing on sequencing and conflict resolution and by the time phase of a normal circadian rhythm. At the ACU, ATCOs manage flights that are mainly on route, causing monotonous monitoring and routine reporting tasks, and the ATCOs generally have frequent night shifts to handle overflights. Their fatigue was significantly influenced by the demand characteristics from tasks, but changes in fatigue rule were not consistent with a normal circadian rhythm, revealing that the ATCOs’ circadian rhythms may have already been slightly disturbed. Furthermore, the interactions between task demand and circadian rhythm with an ATCO’s fatigue were significantly observed in ATCOs working in the TCU but not in those in the ACU. This study provides first evidence that an ATCO’s work type influences his or her fatigue. This discovery may incite stakeholders to consider work type in the management of employee fatigue, not only in the civil aviation industry but also in other transport industries.

## 1. Introduction

The fatigue of air traffic controllers (ATCOs) on duty seriously threatens air traffic safety. Fatigue is a physiological state of reduced mental or physical performance capability resulting from sleep loss, extended wakefulness, circadian phase, and/or workload (mental and/or physical activity) that can impair a person’s alertness and ability to perform safety-related operational duties [1]. Additionally, ATCOs are responsible for preventing collisions between aircrafts, on the maneuvering area between aircraft and obstructions, as well as expediting and maintaining an orderly flow of air traffic [2]. Therefore, if ATCOs become fatigued at work and their alertness is impaired, preventing them from executing their duties, air traffic safety is threatened. For example, in 2011, ATCOs sleeping on duty resulted in flights having to land on their own under dangerous conditions [3]. This phenomenon has been observed previously [4]. In China, a serious runway incursion between two passenger aircrafts occurred at Tongren Fenghuang Airport in October 2017; the investigation report indicated that part of the ability of the ATCO on duty was disturbed by fatigue, causing his alertness and performance to decrease [5]. Recently, increasing attention has been devoted to research on ATCOs’ fatigue [6]. For example, the Federal Aviation Administration of the United States (FAA) delegated the MITRE Corporation to survey the relevant published and current ongoing research on ATCOs’ fatigue [7]. Meanwhile, in 2016, the International Civil Aviation Organization (ICAO) convened a symposium on fatigue management under the theme of “evolution from the cockpit to the air traffic control unit”.

Nonetheless, the fatigue of ATCOs remains a challenge. First, ATCOs at work tend to suffer from fatigue owing to high task demands and heavy pressure. Moreover, their jobs require concomitantly, dealing with multiple types of information (e.g., navigation guidance, weather reports, and emergency alerts), making accurate and rapid decisions, and providing pilots with instructions to ensure a safe and orderly flow of air traffic. Specifically, they need to understand data from a variety of sources, to utilize them to position aircrafts, and to project the positions of each aircraft forward into the near future. Additionally, they are requested to work reliably, efficiently, and effortfully while constantly focused to monitor, control, check, diagnose, and solve problems for a considerably period [8]. Second, to provide services 24/7, ATCOs must work in shifts. The shift work causes them to often have to work at night and to sleep in the day; maintaining regular sleeping is hard as they sometimes wake up early for an early shift and sometimes go to bed late for a night shift. These disturb their circadian rhythms. Therefore, shift work is likely to lead to fatigue. To mitigate ATCOs’ fatigue, the FAA and the National Air Traffic Association (NATA) established a group devoted to optimizing shift work [9]. Therefore, it is generally accepted that ATCOs experience a typical form of mental fatigue [6,10,11], given that their tasks are mentally demanding. Mental fatigue affects alertness, reactive ability, and decision-making power, reduces ATCOs’ performance, and can even threaten traffic safety [12,13,14,15].

**Figure 1:**
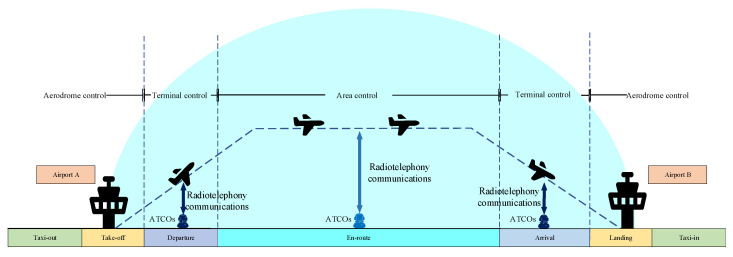
Diagram of control area.

Currently, the division of labor for ATCOs has become increasingly specialized, generating several types of work including aerodrome control, terminal control, and area control (Figure 1). An aerodrome controller mainly focuses on aerodrome traffic, while a terminal or area controller focuses on traffic in the air. Generally, an aerodrome controller is aware of aerodrome traffic situations by looking out of the window from a tower, while a terminal or area controller learns about air traffic situations through screen with an air traffic control automation system in a room. Nowadays, these control modes are very different. An aerodrome controller performs the procedure control with a passive manner, but a terminal or area controller performs the surveillance control with a positive manner. Therefore, it is more comparable between terminal control and area control, which are more similar, considering the particularity of aerodrome control. At a terminal control unit (TCU), ATCOs handle departures and arrivals, which make more convergence with and maneuver around conflicts. Their task demands are more focused on sequencing and conflict resolution. Moreover, ATCOs work less night shifts because red-eye flights are fewer. At the area control unit (ACU), the ATCOs manage flights that are mainly on route, and the task demands lie mainly in monotonous monitoring and routine reporting for longer durations. Note that the ATCOs have to handle overflights that are scheduled as daytime departures or arrivals and have long journeys at night; therefore, ATCOs working at the ACU generally have higher frequent night shifts than those working at the TCU. Considering the different characteristics in the task demand and the circadian rhythm between the two work types, a question arises: does the work type of ATCOs influence their fatigue? To the best of our knowledge, no field studies have been reported on this matter.

This paper presents a cross-sectional field study that is the first to investigate the influence of work type on ATCOs’ fatigue. We selected ATCOs working at the TCU and ACU, and considered both as typical work types. A data-driven detection method using percentage of eyelid closure over the pupil over time (PERCLOS) as an objective and effective measurement method was innovatively employed to characterize the fatigue levels of ATCOs before and after shift work. PERCLOS is a valid psychophysiological measure of fatigue recommended by the Office of Motor Carriers, Federal Highway Administration of the U.S.A. [16]. The Spearman correlation test analysis, a nonparametric test method used to detect trends, is introduced to fatigue studies for the first time. We aim to shed light on ATCOs’ fatigue and to provide new clues to manage employee fatigue within and outside of civil aviation.

## 2. Methods and Experiments

### 2.1. Methods

We conducted a cross-sectional field study in one of the busiest control centers in China because the fatigue of ATCOs is sensitive to heavy pressures to maintain safety [6], and ATCOs do not feel as much pressure to maintain safety in laboratorial or simulator environments.

#### 2.1.1. Selection of Fatigue Detection Method

To detect ATCOs’ fatigue, we adapted the chosen method to the ATCO work environment; hence, the fatigue detection method had to be simple and easy to operate and not a distraction to ATCOs (i.e., to avoid arousing their vigilance and potentially disturbing any measurements of their fatigue status), and its data collection procedure should be rapid (i.e., to reduce ATCOs’ burden of participation). Recently, many items of portable equipment have been developed for the quantification of physiological and biomechanical indices and applied to industrial ergonomic research, including fatigue research [17,18]. After comparing some available fatigue detection methods [19,20,21], we selected the PERCLOS index for this field study because of its high validity, reliability, and noncontact use.

In fact, following the development of computer technologies applicable to image processing, the PERCLOS method became the most widely used measurement tool in the automobile market for detecting driving fatigue [22,23]. Moreover, PERCLOS can measure slow eyelid closures (“droops”) rather than blinks [16]. In order to apply this method to fatigue detection in the field, we employed a laptop with a front-facing camera in the experimental setting and improved the PERCLOS method from a previous work. We previously established the PERCLOS detection method using a multi-task convolutional neural network-convolutional neural network (MTCNN-CNN) model. The model combines a multi-task convolutional neural network for face and eye detection with a convolutional neural network for PERCLOS calculation, and was validated to detect PERCLOS in the field. Details on the detection method can be found in another paper [24]. In order to adapt this method to field studies, we optimized the method with improved robustness, employed a parallel computing mechanism, and used the P70 index as a metric in this study. P70 is defined as the duration when the eyes are closed at least 70%, often employed as a fatigue metric when analyzing PERCLOS data [25]. PERCLOS values, such as P70, increase when subjects become fatigued [25].

#### 2.1.2. Establishment of the PERCLOS Method for Fatigue Measurement

**Figure 2:**
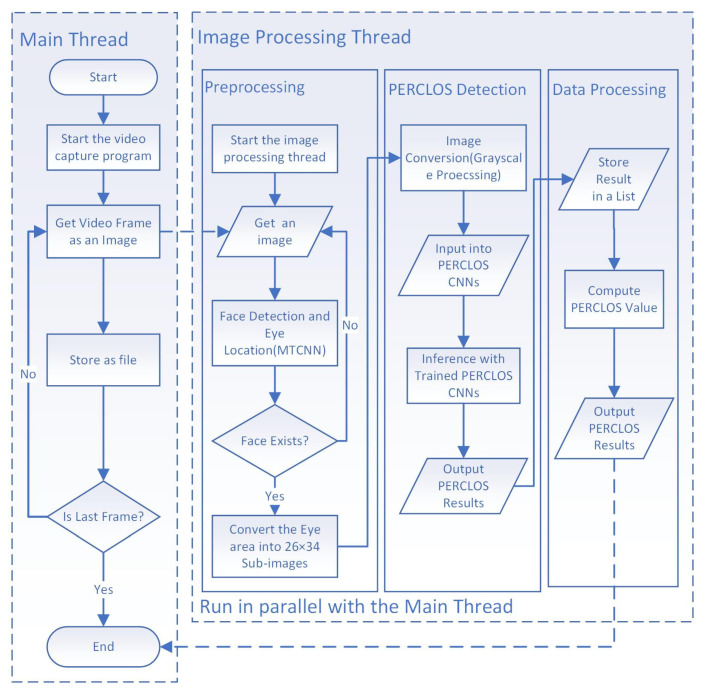
Process of video treatment for detecting air traffic controllers’ PERCLOS values.

To detect the ATCOs’ fatigue levels using the PERCLOS index, we collected image data of the ATCOs’ faces and extracted the PERCLOS values for each image using a computer program employing the MTCNN-CNN model. In brief, the collected image was input into the computer program and the MTCNN model was employed to detect the faces and eyes of the participants. The image was cropped to keep just the eye as the ROI (region of interest), and then, it was converted to sizes of 26 × 34 in order to remove extraneous elements for rapid, accurate computing. Every image of just eyes was converted to grayscale. Then, the CNN model was employed to detect eye status—including calculating the PERCLOS according to the P70 value—attaching either an open or closed PERCLOS status to the image. Different from previous work, the current optimized method mainly had the following two advantages. First, we optimized the detection framework by adding a judgment and jump step in the preprocessing step to improve the robustness of the program. Second, we exploited a parallel computing mechanism to obtain real-time performance, while the process of the previous method included collecting images first and calculating the PERCLOS after. We used one main thread to capture real-time video from the camera. Meanwhile, another thread for image processing was also started and ready to process an image. Each frame of the video was input into the image processing thread and processed while the main thread captured another frame. The overall architecture is shown in Figure 2.

To test the effectiveness of the improvement in robustness, we conducted a pre-experiment with five volunteers to test the face detection rate using a pre-existing method and the current method. The results showed that the face detection rate increased by about 28% for the current improved method (Table 1). The performance from the PERCLOS detection is shown in Table 2.

P70 indicates an eye-lid closure at least 70% [16]. Additionally, the PERCLOS values were calculated as the average percentage of images taken every minute marked with the close status—according to the P70 value as shown in Equation (1).

(1) $$PERCLOS\;value=\frac{\sum_{i=0}^{n}sign\left\{P_{i}\right\}}{FPs\times t}\times 100\%$$

where *Pi* is the rate of a volunteer’s eye being covered at frame *i*, where the value of $\;P_{i}$ is 1 when frame *i* matches the P70 threshold or is 0 when frame *i* does not match the P70 threshold, *sign*{*∙*} is an indicator function, and its value is determined by the rule that *sign*{*True*} = 1, *sign* {*False*} = 0, *FPs* is frames per second, and *t* is test duration.

### 2.2. Experiments

#### 2.2.1. Experimental Design

**Figure 3:**
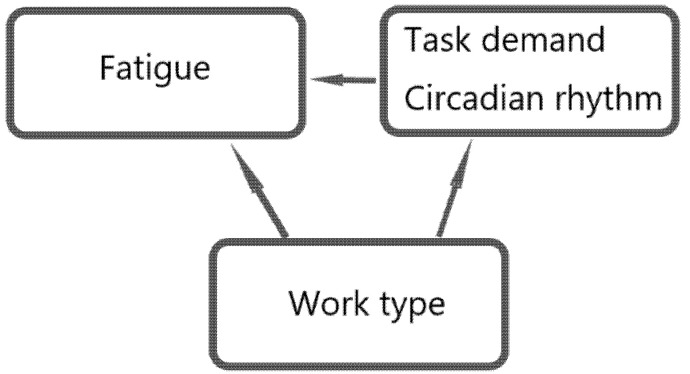
Scheme of the relationship among ATCOs’ fatigue, work type, task demand, and circadian rhythm.

This study employed a 2 × 2 × 4 three-factor mixed design to investigate ATCOs’ fatigue at the shift level (Table 3). ATCOs’ fatigue levels were set as the dependent variable, indexed by the PERCLOS value. Work type, circadian rhythm, and task demand were set as three independent variables (the three factors) (Figure 3).

The first factor, work type, was categorized into two levels: TCU and ACU. In this study, fatigue detection was carried out in the field and ATCOs were not disturbed while working on duty for aviation safety; therefore, fatigue detection was only performed pre- and post-shift. Considering that fatigue is a kind of physiological status and that a person can recover after some rest, fatigue detection was performed just before (pre-) or after (post-) a shift. ATCOs rested before their shift (pre-shift), did not undertake any tasks, and recovered from the fatigue of the last work shift. An ATCO’s fatigue accumulates when they undertake demands from tasks while on duty, and the value of post-shift fatigue reflects the total fatigue from demands of tasks during the whole work shift.

The second factor, task demands, was set at two levels: specifically, the pre-shift level and the post-shift level. The first refers to ATCOs before air traffic control work, in which the demand from tasks is initially low (their fatigue during this pre-shift period was detected just before shift work); the second refers to ATCOs after air traffic control work, in which the demand of tasks is accumulated to a high level (their fatigue during this post-shift period was detected just after shift work). The demands of ATCOs’ tasks for the two work types included monitoring flights, routine reporting, and sequencing and conflict resolution. Work regarding monitoring flights and routine reporting is monotonous and continuous and was parameterized by the mean flight volume per hour, including arrival, departure, and overflight, and by the mean monitoring time per flight. Work regarding sequencing and conflict resolution is complex and urgent and was parameterized by the means for heading, speed, and altitude change per flight, which could partly reflect maneuvering.

The third factor, circadian rhythm, was set at four levels: morning, afternoon, evening, and midnight. To assess the fatigue levels of the ATCOs during different periods, we selected times based on shift changes; specifically, work during 8:00–12:00 was set as shift I morning, 12:00–18:00 was set as shift II afternoon, 18:00–24:00 was set as shift III the first part of night, and 24:00–8:00 was set as shift IV the second part of night. Based on these shifts, we assessed ATCO fatigue in the morning (8:00 ± 30 min), afternoon (12:00 ± 30 min), evening (18:00 ± 30 min), and midnight (24:00 ± 30 min).

#### 2.2.2. Participants

ATCOs working at the TCU and ACU in one of the busiest control centers in China were recruited from August 26 to 30, 2019 (i.e., 5 work days). In total, 69 healthy ATCOs with three class A medical certificates participated after giving their written informed consent. Participants were told that they were free to withdraw from the study at any time. Among the study population, 34 ATCOs worked at the TCU; however, two of these ATCOs participated in the pre-shift detection and did not attend post-shift detection, so their data were excluded from the study. The other 35 worked at the ACU, and all of them completed the detections (Table 4).

According to the Air Traffic Control Rules of China (CCAR-93-R5), ATCOs with fatigue [1] are forbidden from performing control duties. Therefore, all study participants were requested to rest adequately before a shift and to report when they felt that their alertness improved enough to perform their duties, namely, when they perceived that they had recovered from the fatigue experienced during prior shift work.

#### 2.2.3. Fatigue Level Measurement

To collect data, during shift changes, each study participant was asked to face the camera of a personal computer before beginning their shift work for a 5 min test—namely, to collect pre-shift data. To ensure the collection of high-quality videos with full-face photos of the ATCOs, we asked the participants to play a simple game on the computer. The game included letters shown sequentially on the computer screen, and the ATCOs were asked to press the space key when a letter appeared. This operation served to ensure that participants would face the computer camera. After pre-shift data collection, they began their shift normally. Similarly, ATCOs finishing their shifts were requested to perform the same data collection steps before leaving—with the only difference being that the data collected were categorized as post-shift data. The speed of image collection was 24 images per second, which is a common configuration for cameras. Additionally, the PERCLOS values were obtained according to the method presented in Section 2.1.2.

### 2.3. Data Analysis

The PERCLOS values were analyzed using several data analysis methods (Table 5). To the best of our knowledge, the Spearman correlation test is introduced in a fatigue study in the field for the first time. The Spearman correlation test is a classic nonparametric test that uses rank-based inverse normal transformation to conduct nonlinear data transformation, allowing for normalizing data and analysis [26,27,28]. Its null hypothesis is that there is no monotonic association between the two variables in the population, and the alternative hypothesis is that there is a monotonic association between the two variables in the population. It appropriately detected trends and is widely applied to analyze cognitive neuroscientific data, and hydrological time series data, among others [26,27]. The Wilcoxon matched-pairs test is a nonparametric equivalent of the paired *t*-test and is most commonly used to test for differences in the means (or medians) of paired observations [28]. The repeated measures analysis of variance (ANOVA) is used to analyze the variance of subjects measured more than once to determine whether statistically significant changes occurred, for example, from pretest to posttest [28]. The two-sided test was applied to the all statistical analysis test and the result is regarded as significance when *p* value is less than or equal to 0.05 or as very significance when *p* value is less than or equal to 0.01.

## 3. Results and Discussion

### 3.1. Influence of Work Type on ATCOs’ Fatigue

The Spearman correlation test appropriately detects trends between two variables with a time series. In general, the Spearman coefficient *r_s_* value indicates the correlation strength, an absolute value *r_s_* bigger than 0.8 represents a very strong correlation, a value between 0.8 and 0.5 represents a strong correlation, a value between 0.5 and 0.3 represents a weak correlation, and a value less than 0.3 represents a very weak correlation [28]. The very weak correlation is regarded as the two variables being basically irrelevant in a certain confidence interval [28]. Additionally, when the *p* value is bigger than 0.05, its null hypothesis suggesting no monotonic association between the two variables is accepted: the two variables are significantly different within a 95% confidence interval [28].

**Figure 4:**
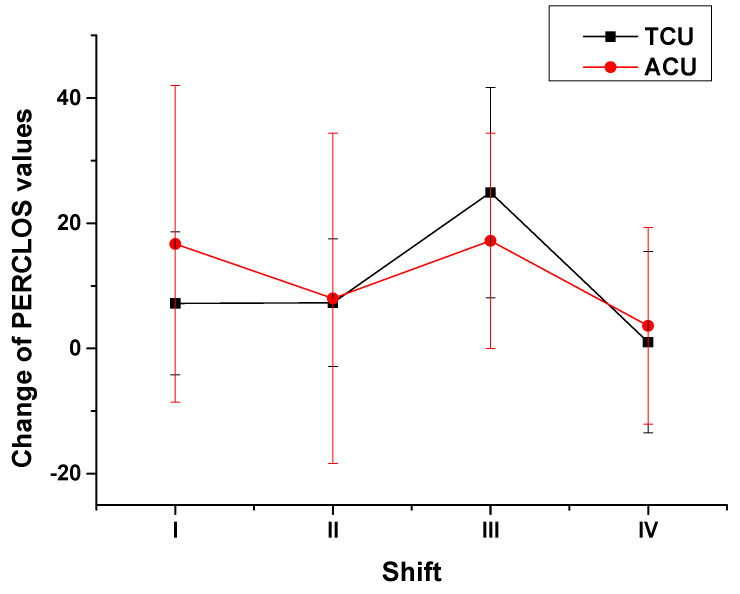
Changes in PERCLOS values of ATCOs by shift and work type.

First, the change in PERCLOS values was defined as the difference: the post-shift values minus corresponding pre-shift values. The variation for the changes in PERCLOS values of ATCOs by shift and work type are shown in Figure 4. For ATCOs at the TCU, their changes in PERCLOS values (Figure 4, black line) were stable from shift I to shift II, increased sharply in shift III, and finally decreased sharply in shift IV. However, for ATCOs at the ACU, their changes in PERCLOS values (Figure 4, red line) decreased from shift I to shift II, increased in shift III and decreased in shift IV. The normality test results showed that the data from neither work type were normally distributed (*p* < 0.001 and *p* < 0.001). The results of the Spearman correlation test between the changes in PERCLOS values of ATCOs at the ACU and those of ATCOs at the TCU with all four shifts showed that the coefficient was only 0.21 (*p* = 0.240). The absolute value of the coefficient was less than 0.3, and *p* was greater than 0.05; therefore, the correlation between the changes in the PERCLOS values of the two work types appeared to be very weak. Accordingly, the null hypothesis was accepted and the alternative hypothesis was rejected, suggesting that a significant difference in the influences of work type on the change in an ATCO’s fatigue was observed.

**Figure 5:**
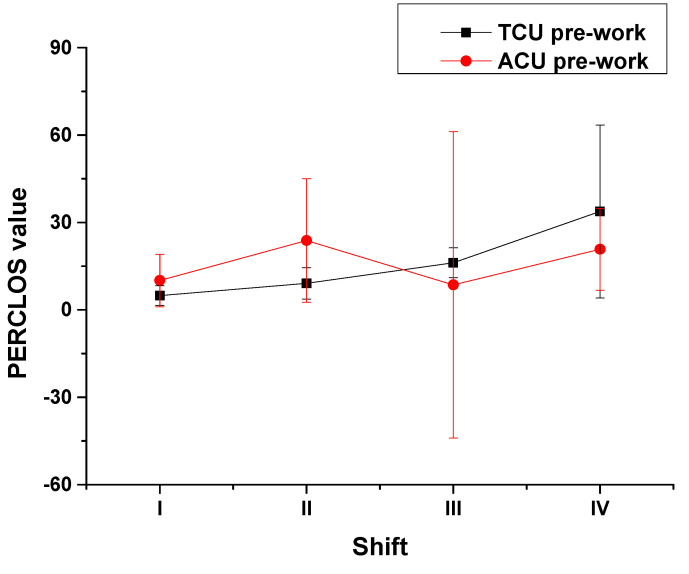
Pre-shift PERCLOS values of ATCOs by shift and work type.

Furthermore, pre-shift fatigue varied differently with work type (Figure 5). For ATCOs at the TCU, their pre-shift PERCLOS values increased from morning to night before work (Figure 5, black line). However, for ATCOs at the ACU, these values fluctuated during the same period, reaching a peak in the afternoon (Figure 5, red line). The curve trends of pre-shift PERCLOS values for the two work types were obviously different. The results of the normality test showed that data from neither work type was normally distributed (TCU: *p* < 0.001; ACU: *p* = 0.001). The Spearman correlation test results between the pre-shift PERCLOS values of ATCOs at the TCU and those of ATCOs at the ACU with all four shifts showed that the coefficient was only 0.15 (*p* = 0.426). Namely, the difference in the influences of work type on an ATCO’s pre-shift fatigue was significant.

**Figure 6:**
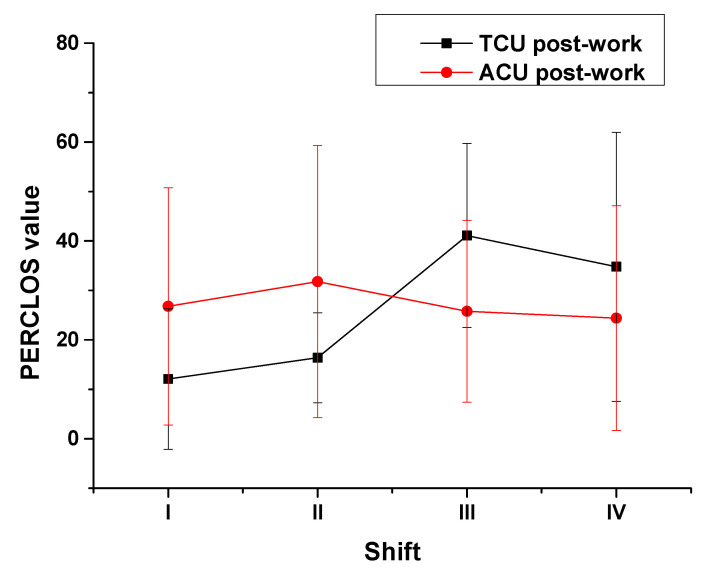
Post-shift PERCLOS values of ATCOs by shift and work type.

Then, the variations in the post-shift fatigue of ATCOs by work type was also investigated. The differences by work type are shown in Figure 6. For ATCOs at the TCU, the post-shift PERCLOS values increased from morning to night, reached a peak during the first half of the night but decreased slightly during the second half of the night (Figure 6, black line); for ATCOs at the ACU, although their post-shift fatigue also fluctuated, the range of fluctuation was lower than that for pre-shift fatigue (Figure 6, red line). The normality test results showed that data from neither work type were normally distributed (*p* = 0.004 and *p* = 0.028). The Spearman correlation test results between the post-shift PERCLOS values of ATCOs at the TCU and those of ATCOs at the ACU with all four shifts showed that the coefficient was 0.03 (*p* = 0.853). Namely, the difference in the influences of work type on an ATCO’s post-shift fatigue was significant.

Summarizing, considering that the sample size of shift IV for both work types were limited, the influence of work type on an ATCO’s fatigue had interesting trends, manifesting in the following indexes: the fatigue changes from pre- to post-shift, as well as pre- and post-shift fatigue separately. These results led to a separate analysis of the PERCLOS data by work type.

### 3.2. Influence of Task Demand on ATCOs’ Fatigue by Work Type

Since the pre- and post-shift PERCLOS values of ATCOs were not normally distributed, we chose to apply a nonparametric test, namely the Wilcoxon matched-pairs test (Table 6). Regarding the TCU, the test results between the pre-shift PERCLOS values and the corresponding post-shift PERCLOS values (*Z* = −3.60; *p* < 0.001) showed that the *Z* value was negative, indicating that the pre-shift PERCLOS values were lower than the post-shift values, and that the *p* value was less than 0.01, indicating that the difference was very significant; specifically, the median was lower by 7.2. Hence, the task demand at the TCU increased an ATCO’s fatigue very significantly. The results were consistent with those of another study using a subjective scale to assess the fatigue of ATCOs at one terminal unit in China [14]. Similarly, we also found a very significant influence of the task demand at the ACU on an ATCO’s fatigue.

We further analyzed the relationship between fatigue increase level and the characteristics of task demand for the two work types. The change in fatigue level was roughly consistent with the change in the characteristics of task demand from shift to shift, as shown in Table 7. The ATCOs’ task demands for the two work types were characterized as the mean flight volume per hour including arrival, departure, and overflight; the mean monitoring time per flight; the means for heading, speed, and altitude changes per flight, which could partly reflect maneuvering. At the TCU, along with the increase in arrival volume triggering most convergences and conflicts, the ATCOs were supposed to bear more task demands and their fatigue levels gradually increased from shift I to shift II and reached a peak in shift III, and decreased in shift IV with the reduction in flight volume. At the ACU, we observed that ATCOs’ fatigue levels were higher when the flight volume was higher, except in shift III. We deduced that the highest arrival volume in shift III also contributed greatly to the peak in fatigue increase because the ATCOs were obligated to help the TCU handle arrival sequencing during busy hours. Comparing the changes in PERCLOS values from the two work types, the median change at the ACU (12.4) was a little higher than that at the TCU (7.2) and reached a significant difference based on the Spearman correlation test (Figure 4). Namely, the task demands at the ACU led to higher fatigue increase levels than that at the TCU as a whole. Those results were related to the monotonic characteristics of task demands at the ACU. At the ACU, task demands were mainly continuous and monotonous monitoring and routine reporting work, and the average monitoring time for per flight reached 21.9 min (Table 7) [29]. Additionally, it was reported in previous works that the monotonous and continuous task demands resulted in ATCOs’ fatigue [15,30]. However, the task demands at the TCU were diversified, and the average monitoring time for per flight was only 11.2, which resulted in reducing the monotonicity of the work. The diversity of task demands at the TCU was beneficial for ATCOs in maintaining alertness and reducing their fatigue. It was worthy to notice that the PERCLOS values of ATCOs at the TCU rapidly increased even higher than the values at the ACU in shift III. That was different with the other three shifts (Figure 4). The heavy pressure of air traffic safety with both higher flight volume and the highest arrival volume resulted in the ATCOs’ fatigue levels at the TCU growing quickly and surmounting the level from the monotony at the ACU. These results indicated that the multiple characteristics of task demand, such as monotonicity, continuity, complexity, and urgency were simultaneously present in field for the two work types, but the prominent characteristics changed with shifts and work types. It is necessary to learn the changes in the characteristics of demands from tasks with shift and work type to manage ATCOs’ fatigue.

Compared with pre-shift PERCLOS values, post-shift values were higher for every shift and work type; this finding was in line with expectations [5,10,11]. Summarizing, the influence of task demands on an ATCO’s fatigue appeared significant trend for both work types on the whole, and the degree of influence was changed along with the characteristics of task demands with different work types and shifts.

### 3.3. Influence of Circadian Rhythm on ATCOs’ Fatigue by Work Type

**Figure 7:**
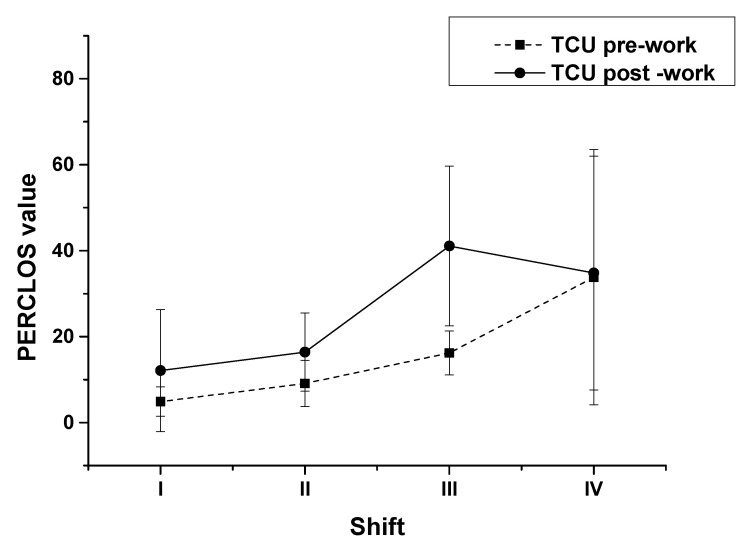
PERCLOS values of ATCOs at the TCU by shift.

Using circadian rhythm as a between group variable, we employed a repeated measures ANOVA to the PERCLOS values of ATCOs at the TCU by shift (Figure 7). The F-test statistically assesses the equality of means of the PERCLOS values for the four shifts (Table 8). The results of PERCLOS values of ATCOs at the TCU for all four shifts including pre- and post-shifts showed that the PERCLOS values were very significantly different by shift (Table 8, F (3, 28) = 7.07, *p* = 0.001). Namely, the influence of circadian rhythm on the fatigue of ATCOs at the TCU was very significant. To determine the influence of circadian rhythm on pre- and post-shift fatigue, we employed one-way ANOVA to analyze the PERCLOS values of ATCOs at the TCU for all four shifts. The results showed that there were very significant differences in the pre-shift values by shift (F (3, 31) = 7.071, *p* = 0.001); namely, the influence of circadian rhythm on the fatigue of ATCOs before work was very significant. We also found very significant differences in the post-shift values by shift (F (3, 31) = 6.049, *p* = 0.003); accordingly, the influence of circadian rhythm on the fatigue of ATCOs after work was very significant. To learn the details of these very significant differences, multiple comparison analysis, using repeated measures by shift, was employed to the PERCLOS values of ATCOs at the TCU. The results of the PERCLOS values of ATCOs at the TCU for all four shifts including pre- and post-shifts showed that the PERCLOS values in shift I were very significantly different from those in shifts III (Table 8, *p* = 0.003) and IV (*p* = 0.001). Moreover, the PERCLOS values in shift II were very significantly different from those in shifts III (*p* = 0.006) and IV (*p* = 0.003). No significant difference was observed between shifts I and II (*p* = 0.431) or between shifts III and IV (*p* = 0.447). Then, we further investigated the tendencies of these significant differences. A multiple comparison analysis of the pre-shift PERCLOS values by shift revealed that, for shift IV, the values were very significantly higher than those in shift I (*p* < 0.001), shift II *(p* < 0.001), and, obviously, those in shift III (*p* = 0.015). There was no significant difference between shifts I, II, and III. Thus, ATCOs’ fatigue fluctuated smoothly from 8:00–18:00 (i.e., daytime) and significantly increased in the second half of the night. This changing curve coincides with the timing of cortisol production in a general circadian rhythm; based on prior research, cortisol production remains stable during the daytime and decreases significantly at night, and during this decrease, melatonin secretion (another circadian rhythm related secretion) begins to increase, leading to sleepiness [24,29,30]. Then, we employed a multiple comparison analysis for post-shift PERCLOS values by shift. The results differed from those for pre-shift values. For shift III, the PERCLOS values were very significantly and significantly higher than those in shifts I (*p* = 0.001) and II (*p* = 0.026), respectively. Meanwhile, the PERCLOS values in shift IV were very significantly higher than those in shift I (*p* = 0.002) and significantly in shift II (*p* = 0.044). No significant difference was observed between shifts I and II (*p* = 0.546) or between shifts III and IV (*p* = 0.516). These results were similar to those of another study on ATCOs’ fatigue at one terminal unit in China, which used the Stanford Sleepiness Scale [14].

**Figure 8:**
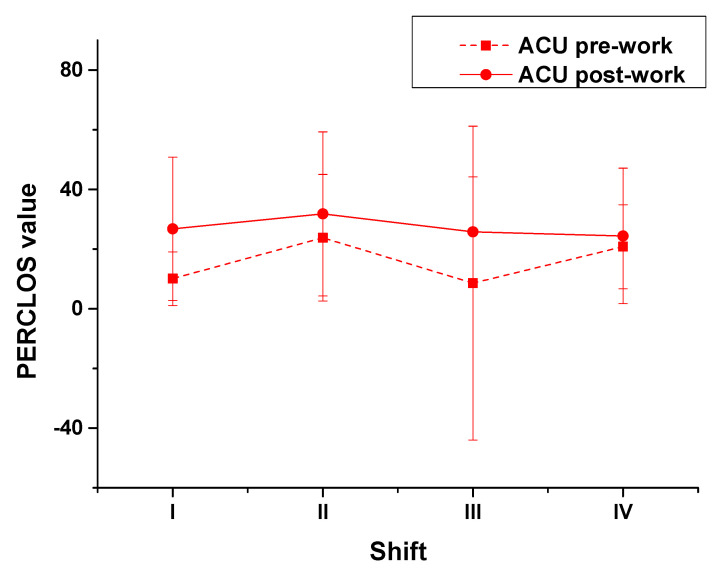
PERCLOS values of ATCOs at the ACU by shifts.

We also investigated the influence of circadian rhythm of ATCOs on fatigue at the ACU using the same analytical steps as that for TCU (Figure 8). The results of the repeated measures ANOVA (with circadian rhythm as a between group variable) of the PERCLOS values of ATCOs at the ACU for all four shifts including pre- and post-shifts showed that the PERCLOS values did not differ significantly by shift (Table 8, F (3, 31) = 0.99, *p* = 0.408). Namely, the influence of circadian rhythm was not significant. To determine the influence of circadian rhythm on pre- and post-shift fatigue at the ACU, the results of the one-way ANOVA showed that the influence of circadian rhythm on the fatigue of ATCOs before work was significant (F (3, 34) = 3.145, *p* = 0.039). Regarding post-shift, notwithstanding, there was no significant difference (F (3, 34) = 0.165, *p* = 0.919). That is, the influence of circadian rhythm on the fatigue of ATCOs after work was not significant in the ACU context; hence, post- and pre-shift fatigue were influenced differently by circadian rhythm. Compared with the results of the repeated measures ANOVA (which used circadian rhythm as a between group variable), the changes in ATCOs’ post-shift fatigue (presented in the one-way ANOVA results) were consistent with those of the results of the repeated measures of variance analysis between groups and different shifts in pre-shift fatigue. To learn the details of the changes in ATCOs’ fatigue, the results of the multiple comparison analysis for the PERCLOS values of ATCOs at the ACU for all four shifts including pre- and post-shifts, using repeated measures by shift, showed that there was no significant difference between the shifts (Table 8). We further analyzed the influence of circadian rhythm on pre- and post-shift fatigue. The multiple comparison analysis for pre-shift PERCLOS values by shift revealed that, for shift II, the values were significantly higher than those in shift I (*p* = 0.046) and very significantly higher than those in shift III (*p* = 0.010); however, no significant difference was observed between shifts II and IV (*p* = 0.679). The difference between the two work types may be related to the different frequencies of night shifts. ATCOs at the TCU worked night shifts (shift IV) once a month, while ATCOs at the ACU worked night shifts once every 12 days. This changing curves of ATCOs at the ACU did not coincide with a normal circadian rhythm, implying that their circadian rhythm may be slightly disturbed. Hence, ATCOs differed in their circadian rhythm by work type, as those at the TCU were shown to maintain a normal circadian rhythm while those at the ACU did not maintain it. A multiple comparison analysis was employed to test the post-shift PERCLOS values at the ACU by shifts. The results differed from those for pre-shift; the post-shift values were not significantly different by shift.

Summarizing, the change in ATCOs’ fatigue observed following the shifts from morning to night was roughly ascribed to circadian phases. The influence of circadian rhythm on ATCOs’ fatigue differed by work type. At the TCU, ATCOs maintained a normal circadian rhythm, with fatigue levels reaching a peak at night, and the influence of circadian rhythm appeared to be significant; at the ACU, the circadian rhythm of ATCOs may have been slightly disturbed, showing two peaks in the fatigue levels.

### 3.4. Interaction between Task Demand and Circadian Rhythm and its Influence on ATCOs’ Fatigue by Work Type

For ATCOs at the TCU, a multiple-factor repeated measures ANOVA was employed for the pre- and post-shift PERCLOS values by shift (Table 9). The results showed that the interaction effect appeared to be significant (F (3, 28) = 4.13; *p* = 0.015). This result was consistent with that of another study using a questionnaire [15]. For ATCOs at the ACU, a similar analysis showed that the interaction effect was not significant.

## 4. Conclusions

ATCOs’ fatigue must be managed because it threatens air traffic safety. Researchers have attempted to optimize the work schedule of ATCOs, limiting their workloads [31]. Meanwhile, the ICAO issued guidelines to limit their work/rest time. However, ATCOs can perform several different types of work, with each work type having different characteristics. The different characteristics of these work types result in different task demands and circadian rhythms. The differences in task demands and circadian rhythms may lead to different ATCO fatigue. This study was the first cross-sectional field study to investigate the influence of work types on ATCOs’ fatigue using the data-driven PERCLOS detection method. Using well-designed experiments and several skillful statistical analysis methods with multi-perspectives, several preliminary attractive discoveries were obtained.

The findings showed that the influence of work types on ATCOs’ fatigue had interesting trends. Specially, ATCOs at the TCU who handle departures and arrivals, which make more convergence with and maneuvers around conflicts, maintain a normal circadian rhythm because of the fewer night shifts as few red-eye flights are fewer. The results showed that their fatigue appeared to be significantly influenced by various task demands focusing on sequencing and conflict resolution and by the time phase of a normal circadian rhythm. At the ACU, ATCOs handle on-route flights, deal with monotonous monitoring and routine reporting tasks for a longer duration, and generally have higher frequent night shifts to handle overflights, which are scheduled as daytime departures or arrivals and have long journeys at night. The results showed that their fatigue appeared to be significantly influenced by the characteristics of the task demands, but the change in fatigue was not consistent with a normal circadian rhythm, revealing that ATCOs’ circadian rhythms may have already been slightly disturbed. Furthermore, the interaction between the task demands and circadian rhythm with an ATCO’s fatigue was significantly observed at the TCU but not at the ACU. This evidence for different variations in ATCO fatigue by work type are conducive to the development of targeted management plans for dealing with the fatigue of these professionals; namely, targeted management should be applied instead of one-size-fits-all solutions.

In this study, an optimized data-driven fatigue detection method was established and was employed to detect PERCLOS based on previous work. The robustness was improved by adding a new step to the preprocessing module, and a parallel computing mechanism was applied to obtain real-time performance. The experiments showed that the program was successfully applied to ATCOs in a field study. The optimization made the data-driven method contactless, rapid, and convenient for field studies in complex and undisturbed work environments. Furthermore, the Spearman correlation test analysis was applied to a fatigue study for the first time in this study. The analysis method appropriately assesses the significance of trends in complex and subtle fatigue time series.

Therefore, in this study a new paradigm was established for common fatigue research by introducing the data-driven modified PERCLOS method for the measurement of fatigue and the Spearman correlation test analysis. The paradigm provides a way to study human fatigue in the field and/or for a long time series from a macroscopic point of view. We believe that the paradigm will help to open new perspective on fatigue study evolution in time series in many other 24/7 industries besides civil aviation, such as road and rail transportation, healthcare systems, and manufacturing.

In fact, fatigue has recently become a research hotspot because of its threat to safety and hazards to occupational health [31,32]. This study provides data-driven evidence that work type influences an ATCO’s fatigue. The discoveries from the current study may suggest to stakeholders that the development of strategies for fatigue management may benefit from and be made more effective by learning the relationships between employee fatigue and work types or job characteristics in other industries. However, our findings in this study focused on strategic and indicative problems. Future studies should recruit more subjects to confirm the discoveries in this study. Additionally, the studies in the future should investigate the influence of the characteristics of work types on task demand and circadian rhythm more in depth, employ other methods for fatigue detection in professionals who work shift, and, then, establish a series of function expressions based on work types to describe the relationships between fatigue, task demand, and circadian rhythm. For instance, we described ATCOs’ task demands and found some influence on their fatigue at the shift level, but more dynamic and refined depicting on the task demands from hour to hour, need to be presented. Since we found two peaks in the fatigue levels at the ACU during their circadian phases, how can we explain the disturbance in their circadian rhythm? A deeper understanding of the relationship between the three factors will help improve fatigue management more accurately and more effectively.

## Figures and Tables

**Table 1:** Comparison of the effectiveness for the face detection between previous and current method.

Sample No.	Total Frames	Previous Method Detection Numbers	Previous Method Detection Rate	Current Method Detection Numbers	Current Method Detection Rate
1	14,521	9494	65.38%	13,494	92.93%
2	14,521	11,062	76.18%	12,577	86.61%
3	14,521	10,102	69.57%	14,250	98.13%
4	14,521	8953	61.66%	13,053	89.89%
5	14,521	9903	68.20%	13,344	91.89%
average	14,521	9494	65.38%	13,494	92.93%

**Table 2:** Performance from the PERCLOS detection.

Name	Speed of Image Collection	Frame	Date Bit Rate	Frame Width	Frame Height
Value	24/second	24 FPS/second	14 Mbps/second	640 pixel	480 pixel

**Table 3:** Description of the 2 × 2 × 4 three-factor mixed experimental design.

Factors	Levels			
Work type	TCU		ACU	
Task demand	Pre-shift		Post-shift	
Circadian rhythm	Shift I	Shift II	Shift III	Shift IV

**Table 4:** General information of the ATCO participants.

Work Type	Age	Gender	People Number	Shift I	Shift II	Shift III	Shift IV
TCU	23–36 Mean 29	Male	32	7	14	7	4
ACU	23–38 Mean 29	Male	35	7	9	14	5
Total	--	--	67	14	23	21	11

**Table 5:** Data analysis methods of the PERCLOS values.

Factors	PERCLOS Value Index	Data Analysis Methods
Work type	change, pre-shift, and post-shift	Spearman correlation test
Task demand	change	Wilcoxon matched-pairs test
Circadian rhythm	pre-shift	Repeated measures analysis of variance (ANOVA), One-way ANOVA, and multiple comparison analysis
Interaction between circadian rhythm and task demand	pre- and post-shift	Multiple-factor repeated measures ANOVA

**Table 6:** Comparing task demand influence on the PERCLOS values by work types.

Statistical Method	TCU	ACU
Wilcoxon matched-pairs test	*Z* = −3.60, *p* < 0.001	*Z* = −3.09, *p* = 0.002

**Table 7:** Comparing task demands and ATCOs’ fatigue changes in the TCU and ACU.

Task Demand		Shift I		Shift II		Shift III		Shift IV	
		TCU	ACU	TCU	ACU	TCU	ACU	TCU	ACU
Flight volume	arrival	10	10	26	26	28	28	8	8
	departure	40	40	26	26	22	22	12	12
	overflight	5	164	6	143	5	126	1	28
Monitoring time (min)		10.0	23.0	10.8	22.8	11.6	21.1	12.3	20.7
Heading change		2.2	2.2	2.5	2.0	2.3	1.7	1.9	1.4
Speed change		1.2	0.5	1.0	0.5	1.0	0.5	1.1	0.4
Altitude change		1.1	1.5	1.0	1.4	1.2	1.3	0.8	1.1
Change PERCLOS value		7.2	16.7	7.3	8	24.9	17.2	1	3.6

**Table 8:** Comparisons of the influence of circadian rhythm on ATCOs’ PERCLOS values by work type.

Statistical Method	TCU	ACU
Repeated measures ANOVA	Among the shifts **, F (3, 28) = 7.07, *p* = 0.001	Among the shifts, F (3, 31) = 0.99, *p* = 0.408
One-way ANOVA	Pre-shift **, F (3, 31) = 7.071, *p* = 0.001 Post-shift **, F (3, 31) = 6.049, *p* = 0.003	Pre-shift *, F (3, 34) = 3.145, *p* = 0.039 Post-shift, F (3, 34) = 0.165, *p* = 0.919
Multiple comparison analysis using repeated measures	Shift I vs. shift III **, *p* = 0.003; vs. shift IV **, *p* = 0.001 Shift II vs. shift III **, *p* = 0.006; vs. shift IV **, *p* = 0.003 Shift I vs. shift II, *p* = 0.431 Shift III vs. shift IV, *p* = 0.447	No significant difference by shift was observed.
Multiple comparison analysis for pre-shift values	Shift IV vs. shift I **, *p* < 0.001; vs. shift II **, *p* = 0.001; vs. shift III *, *p* = 0.015 No significant difference observed between shift I, II, III.	Shift II vs. shift I *, *p* = 0.046; vs. shift III *, *p* = 0.010; No significant difference was observed between shifts I, III, and IV.
Multiple comparison analysis for post-shift values	Shift I vs. shift III **, *p* = 0.001; vs. shift IV *, *p* = 0.026 Shift II vs. shift III **, *p* = 0.002; vs. shift IV *, *p* = 0.044 Shift I vs. shift II, *p* = 0.546 Shift III vs. shift IV, *p* = 0.516	No significant difference between the shifts was observed.

* Significant difference, ** very significant difference.

**Table 9:** Comparisons for the influence of the interaction between task demand and circadian rhythm on PERCLOS values by work type.

Statistical Method	TCU	ACU
Multiple-factor repeated measures ANOVA	F (3, 28) = 4.13; *p* = 0.015	F (3,31) = 0.72, *p* = 0.546
